# Assessment of aortic valve area on cardiac computed tomography in symptomatic bicuspid aortic stenosis: Utility and differences from Doppler echocardiography

**DOI:** 10.3389/fcvm.2022.1035244

**Published:** 2022-12-19

**Authors:** Kyu Kim, Soo Ji Lee, Jiwon Seo, Young Joo Suh, Iksung Cho, Geu-Ru Hong, Jong-Won Ha, Young Jin Kim, Chi Young Shim

**Affiliations:** ^1^Division of Cardiology, Department of Internal Medicine, Severance Cardiovascular Hospital, Yonsei University College of Medicine, Seoul, Republic of Korea; ^2^Department of Radiology, Severance Hospital, Yonsei University College of Medicine, Seoul, Republic of Korea

**Keywords:** bicuspid aortic stenosis, aortic valve area, cardiac computed tomography, echocardiography, multimodal imaging

## Abstract

**Background:**

In this study, we investigate the utility of geometric orifice area (GOA) on cardiac computed tomography (CT) and differences from effective orifice area (EOA) on Doppler echocardiography in patients with bicuspid aortic stenosis (AS).

**Methods:**

A total of 163 patients (age 64 ± 10 years, 56.4% men) with symptomatic bicuspid AS who were referred for surgery and underwent both cardiac CT and echocardiography within 3 months were studied. To calculate the aortic valve area, GOA_CT_ was measured by multiplanar CT planimetry, and EOA_Echo_ was calculated by the continuity equation with Doppler echocardiography. The relationships between GOA_CT_ and EOA_Echo_ and patient symptom scale, biomarkers, and left ventricular (LV) functional variables were analyzed.

**Results:**

There was a significant but modest correlation between EOA_Echo_ and GOA_CT_ (*r* = 0.604, *p* < 0.001). Both EOA_Echo_ and GOA_CT_ revealed significant correlations with mean pressure gradient and peak transaortic velocity, and the coefficients were higher in EOA_Echo_ than in GOA_CT_. EOA_Echo_ of 1.05 cm^2^ and GOA_CT_ of 1.25 cm^2^ corresponds to hemodynamic cutoff values for diagnosing severe AS. EOA_Echo_ was well correlated with the patient symptom scale and log NT-pro BNP, but GOA_CT_ was not. In addition, EOA_Echo_ had a higher correlation coefficient with estimated LV filling pressure and LV global longitudinal strain than GOA_CT_.

**Conclusion:**

GOA_CT_ can be used to evaluate the severity of bicuspid AS. The threshold for GOA_CT_ for diagnosing severe AS should be higher than that for EOA_Echo_. However, EOA_Echo_ is still the method of choice because EOA_Echo_ showed better correlations with clinical and functional variables than GOA_CT_.

## Introduction

Aortic stenosis (AS) is a common heart valve disease in an aging society, and its incidence is continuously increasing (1, 2). For several decades, aortic valve (AV) area estimations by Doppler echocardiography have been the gold standard for the assessment of AS severity (3, 4). However, since assumptions and measurement errors affect each component used for calculating the effective orifice area (EOA) with the continuity equation, EOA on echocardiography (EOA_Echo_) has been criticized for primarily underestimating and sometimes overestimating the actual stenotic area (5).

In the era of transcatheter intervention, structural evaluation through cardiac computed tomography (CT) is actively performed when planning surgery or intervention in patients with symptomatic AS and has been established as a principal examination (6). By direct measurement of CT scans, it is possible to obtain the geometric orifice area (GOA_CT_) of stenotic aortic valves (7). Therefore, in real clinical practice, there is often a difference between EOA_Echo_ and GOA_CT_ in the same patient. In particular, the discrepancy between EOA_Echo_ and GOA_CT_ is greater in patients with bicuspid AS compared with those with tricuspid AS (3, 8). Previous studies have shown different flow gradient patterns, greater jet eccentricity, and less pressure recovery in patients with bicuspid AS (3, 8). However, data to determine the utility of GOA_CT_ on assessment for AS severity are scarce, and there are no studies focusing on bicuspid AS. Therefore, in this study, we investigate the utility of GOA_CT_ and differences from EOA_Echo_ in bicuspid aortic stenosis (AS).

## Methods

### Study population

Using a retrospective and prospective registry that consisted of 1,497 patients with BAV of over 19 years, (9). we identified 163 cases of symptomatic bicuspid AS who underwent transthoracic echocardiography and cardiac CT concomitantly (<3 months between examinations). The registry contains echocardiographic data, laboratory findings, and clinical information from medical records of patients in Severance Cardiovascular Hospital, Seoul, Korea. We excluded patients without symptoms related to AS (dyspnea on exertion, syncope, or chest pain), with endocarditis, with at least moderate dysfunction in mitral, tricuspid, or pulmonic valves, and with previous valve repair or replacement. Combined aortic regurgitation (AR) was identified when the patients had at least moderate AR. For determining clinical and laboratory data, including New York Heart Association (NYHA) class and N-terminal pro-b-type natriuretic peptide (NT-proBNP), information at the time closest to the time of echocardiography and CT was used. Natural log-transformed NT-proBNP values were used due to skewed distributions. Each patient was treated according to the clinical judgment of the physician based on the AS treatment guidelines at the time of diagnosis (10, 11). The Institutional Review Board of Severance Hospital approved this study, which was conducted in compliance with the Declaration of Helsinki. The requirement for informed consent was waived for patients included in the data collection of the retrospective registry and was obtained for the prospective data.

### Transthoracic echocardiography

All echocardiographic studies were performed using commercially available equipment and were reviewed by experienced sonographers and imaging cardiologists without knowledge of the clinical data. Standard measurements were performed according to current guidelines (12). Bicuspid AV was diagnosed when only two cusps were unequivocally identified in systole and diastole in the short-axis view, as previously described (8, 9, 13). In all patients, bicuspid AV was confirmed on cardiac CT and echocardiography. Bicuspid AV morphology was classified into the following four types according to the position and pattern of the raphe and cusps, as previously described: type 1: one raphe with a fusion of the left coronary and right coronary cusps; type 2: one raphe with a fusion of the right coronary and non-coronary cusps; type 3, one raphe with a fusion of the left coronary and non-coronary cusps; and type 0: no raphe with two developed cusps (14).

**Figure 1:**
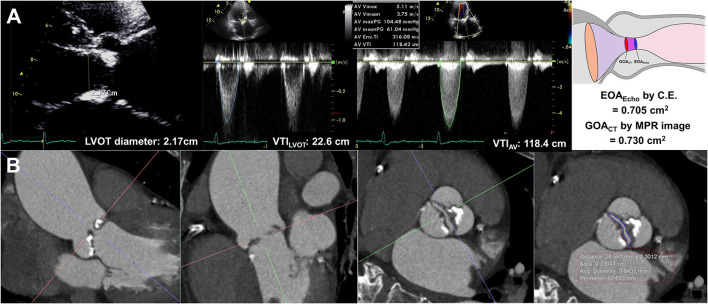
Effective orifice area by continuity equation **(A)** and geometric orifice area by multiplanar computed tomography image **(B)**.

EOA_Echo_ was derived from the continuity equation. The left ventricular outflow tract (LVOT) flow was assessed on the apical three-chamber view. The LVOT diameter was measured during mid-systole 0.5–1 cm below the aortic annulus on the parasternal long-axis view. The velocity–time integral of the LVOT (VTI_LVOT_) was assessed using pulse-wave Doppler. Assuming the circular geometry of the LVOT, the stroke volume was calculated using the following formula: LVOT diameter^2^ × 0.785 × VTI_LVOT_. The highest peak velocity across the AV, mean pressure gradient (MPG), and velocity-time integral of the AV (TVI_AV_) were measured from multiple windows using continuous-wave Doppler (Figure 1). All measurements represent an average of three cardiac cycles for patients with sinus rhythm and an average of five cardiac cycles for patients with atrial fibrillation. The severity of AS and combined aortic regurgitation (AR) was assessed based on the current guidelines (15, 16).

We calculated LV mass using the corrected American Society of Echocardiography method (17). Relative wall thickness was calculated as follows: (2 × posterior wall thickness/LV end-diastolic dimension). LV ejection fraction was measured using Simpson's biplane method (12). The mitral inflow velocities were recorded with pulsed wave Doppler with the sample volume placed at the tip of the mitral valve from the apical four-chamber views. Tissue Doppler imaging was used to measure mitral annular velocities at the septal corner of the mitral annulus. LV filling pressure was estimated by the early diastolic mitral inflow velocity (*E*)/early diastolic mitral annular velocity (*e*') ratio (18). LV global longitudinal strain (GLS) was measured with a two-dimensional speckle tracking analysis on apical two-, three-, and four-chamber views using a vendor-independent software package (TomTec Imaging System; 2D Cardiac Performance Analysis), as described earlier (19). |LV GLS| was defined as the absolute value of LV GLS (removing the conventional negative value of GLS data).

### Cardiac CT measurements

All CT scans were performed with a dual-source CT scanner (SOMATOM Definition Flash; Siemens Healthcare, Forchheim, Germany). Before CT angiography, a non-enhanced prospective ECG-gated scan was performed to measure aortic valve calcium score with standardized calcium scan parameters (slice collimation of 0.6 mm, slice width of 3.0 mm, tube voltage of 120 kV, and a tube current of 50 mAs). CT angiography was performed in retrospective ECG-gated data acquisition mode using the triple-phase injection method (70 ml of iopamidol followed by 30 ml of 30% blended iopamidol with saline and 20 ml of saline at 5 ml/s). Images were generated using iterative reconstruction (sinogram-affirmed iterative reconstruction). Image reconstruction was performed with a medium kernel (I36f), and the reconstruction slice thickness was 0.75 mm with 0.5–mm increments. For all patients, 10 transverse data sets were reconstructed every 10% of the cardiac cycle. Image analysis was performed using 3D software (Aquarius iNtuition, Ver. 4.4.11, TeraRecon, San Mateo, CA, USA). For planimetry, the image volume was rotated into a plane perpendicular to the LVOT and aortic root (20). LVOT imaging involved the orientation of a cross-sectional plane of the LVOT using three orthogonal planes from multiplanar reconstruction at or immediately under the lowest implantation base of the aortic cusp, and two orthogonal diameters (minimal and maximal) were measured (4). GOA_CT_ was defined as the CT planimetry-derived AV area. Planimetry of the AV area was performed at the level of the aortic leaflet tips in the mid-systolic phase, which provided the best visualization of the open aortic valve, usually at 20–30% of the R-R interval, regardless of BAV types (Figure 1, Supplementary Figure 1). Planimetry of the LVOT was performed immediately below the AV in the same phase used for the measurement of the AV area. The angle between the LVOT-AV and aorta (°) was measured during the mid-systolic phase. The AV calcium score was measured using commercial software (Aquarius iNtuition, Ver. 4.4.11, TeraRecon, San Mateo, CA, USA). Observers segmented the AV calcium score by carefully including the region of interest in the AV leaflet and annulus and excluding calcium in the adjacent sinus of Valsalva, left ventricular outflow tract, or mitral annulus, and image noise or beam hardening artifact was excluded. From the segmented ROI, the AVC volume was measured, and an AVC score was calculated using the Agatston method (21, 22). All CT analyses were independently performed by two radiologists blinded to clinical information, echocardiographic results, and CT analysis results of the other reader. Intraclass correlation coefficients (ICCs) were calculated to assess the inter- and intra-observer variability of the GOA_CT_. A total of 20 samples were assessed by the same observer on different occasions in random order and were also assessed by another observer.

### Statistical analysis

The results are expressed as mean ± standard deviation or number (percentage). Differences between EOA_Echo_ and GOA_CT_ were analyzed by paired *t*-test. Correlations and agreements between EOA_Echo_ and GOA_CT_ were assessed by Pearson's correlation and Bland–Altman's methods. Correlations between echocardiographic and clinical parameters and EOA_Echo_ or GOA_CT_ were determined by Pearson's correlation. Correlations between NYHA functional class and EOA_Echo_ or GOA_CT_ were assessed using Kendall's coefficient of rank correlation. Linear regression was used to generate the fitted line for data pairs of EOA_Echo_ and GOA_CT_. Non-linear regression was used to generate the fitted curve for data pairs of conventional Doppler and clinical parameters (MPG, peak flow velocity, NYHA class, log-transformed NT-proBNP, *E*/*e*', and LV GLS) and EOA_Echo_ or GOA_CT_. Correlation coefficients between EOA_Echo_ and GOA_CT_ were compared using Fisher's Z transformation. Subgroup analysis was performed according to the presence of combined significant AR and LV systolic dysfunction (LVEF <50%). A *p*-value of < 0.05 was considered statistically significant. Statistical analyses were conducted using R version 4.1.0 (The R Foundation for Statistical Computing; www.R-project.org).

## Results

### Baseline characteristics

Table 1 shows the baseline characteristics of the study population. The mean age was 64.3 ± 9.6 years, and 92 (56.4%) were male patients. In terms of comorbidities, 88 (53.9%) had hypertension, 36 (22.1%) had diabetes mellitus, and 21 (12.9%) had atrial fibrillation. Type 0 (no-raphe) was the most common BAV phenotype in our study population, followed by Type 1 (RCC+LCC fusion). The average log-transformed NT-proBNP was 6.14 ± 1.50 pg/ml. All patients were symptomatic, and NYHA class II dyspnea was the most common symptom. All patients without dyspnea (NYHA class I) had other AS cardinal symptoms such as chest pain or syncope. The baseline characteristics were not significantly different between patients with concordant and discordant AS degrees (Supplementary Table 1).

**Table 1:** Baseline characteristics of the study population.

	***N*** **= 163**
Age, years	64.3 ± 9.6
Male sex, *n* (%)	92 (56.4)
Body mass index, g/m^2^	24.6 ± 5.0
Hypertension, *n* (%)	88 (53.9)
Diabetes mellitus, *n* (%)	36 (22.1)
Coronary artery disease, *n* (%)	19 (11.7)
Dyslipidemia, *n* (%)	51 (31.3)
Chronic kidney disease, *n* (%)	4 (2.5)
Atrial fibrillation, *n* (%)	21 (12.9)
Prior cerebrovascular accident, *n* (%)	1 (0.6)
Systolic blood pressure, mmHg	122 ± 13
Diastolic blood pressure, mmHg	76 ± 11
Pulse pressure, mmHg	46.0 ± 11.0
**Bicuspid AV morphology**
Type 1 (RCC+LCC), *n* (%)	65 (39.9)
Type 2 (RCC+NCC), *n* (%)	14 (8.6)
Type 3 (LCC+NCC), *n* (%)	3 (1.8)
Type 0 (No raphe), *n* (%)	81 (49.7)
Log NT-proBNP, pg/ml	6.14 ± 1.50
**NYHA class**, ***n*** **(%)**	
I	43 (26.4)
II	85 (52.1)
III	22 (13.5)
IV	13 (8.0)
**Type of surgery performed**	
Aortic valve replacement or aorta replacement	149 (91.4)
Aortic valve replacement	145 (89.0)
Aorta replacement	42 (25.8)

RCC, right coronary cusp; LCC, left coronary cusp; NCC, non-coronary cusp; NT-proBNP, N-terminal pro brain natriuretic peptide; NYHA, New York Heart Association.

**Figure 2:**
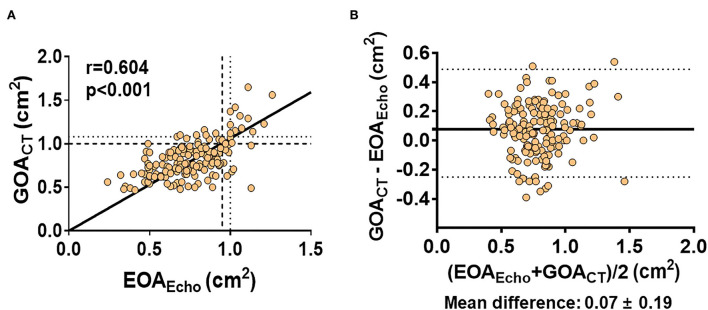
**(A,B)** Correlation between EOA_Echo_ and GOA_CT_. EOA_Echo_ 1.0 cm^2^ correspond to GOA_CT_ of 1.08 cm^2^ (dotted line). GOA_CT_ 1.0 cm^2^ corresponded to EOA_Echo_ of 0.95 cm^2^ (dashed line).

Table 2 presents measurement data from echocardiography and cardiac CT. The mean LVEF was 64 ± 12%, and the mean |LV GLS| was 16.3 ± 4.0%. The average LA volume index was 40.5 ± 15.0 ml/m^2^ and that of *E*/*e*' was 15.7 ± 6.1. The mean EOA_Echo_ was 0.76 ± 0.20 cm^2^. According to EOA criteria, in 147 (90.2%) patients, the AV area was < 1.0 cm^2^, indicating severe AS. In the values measured through cardiac CT, the average GOA_CT_ was 0.83 ± 0.22 cm^2^, which was significantly higher than EOA_Echo_ (mean difference: 0.07 ± 0.19 cm^2^, *p* < 0.001). There was a significant but modest correlation between EOA_Echo_ and GOA_CT_ (*r* = 0.604, *p* < 0.001; Figure 2). The correlations were significant in both sinus rhythm (*r* = 0.555, *p* < 0.001) and atrial fibrillation (*r* = 0.783, *p* < 0.001, *p* for interaction=0.088). The correlations were also significant in BAV type 0 (*r* = 0.494, *p* < 0.001) and other types (*r* = 0.647, *p* < 0.001, *p* for interaction = 0.152). When the diagnostic criteria for severe AS through GOA were applied to < 1.0 cm^2^, the number of patients corresponding to severe AS was 136 (83.4%), which was reduced compared to the number of patients identified by EOA_Echo_ as having severe AS. That is, there were 19 (11.7%) patients with discrepant AS severity evaluated by EOA_Echo_ and GOA_CT_. The inter- (ICC = 0.984; *P* < 0.001) and intra-observer variabilities (ICC = 0.976, *P* < 0.001) for the GOA_CT_ showed excellent agreement.

**Table 2:** Echocardiography and CT measurements.

	***N*** **= 163**
**Echocardiographic data**	
LVEDD, mm	50.0 ± 6.7
LVESD, mm	33.3 ± 7.3
LVEF, %	64 ± 12
LVEF < 50%, *n* (%)	22 (13.0)
|LV GLS|, %	16.3 ± 4.0
LVOT diameter, mm	22.3 ± 2.1
Stroke volume index, ml/m^2^	48.4 ± 10.2
LV mass index, g/m^2^	137.4 ± 40.4
Relative wall thickness	0.47 ± 0.08
LA volume index, ml/m^2^	40.5 ± 15.0
*E/e'*	15.7 ± 6.1
Mean transaortic pressure gradient, mmHg	56.3 ± 18.4
Peak transaortic velocity, m/s	4.69 ± 0.69
EOA_Echo_, cm^2^	0.76 ± 0.20
Indexed EOA_Echo_, cm^2^/m^2^	0.45 ± 0.12
EOA derived severe AS, *n* (%)	147 (90.2)
**CT data**	
GOA_CT_, cm^2^	0.83 ± 0.22
Indexed GOA_CT_, cm^2^	0.49 ± 0.13
LVOT-AV-Aorta angle	16.8 ± 7.9
LVOT average diameter, mm	25.3 ± 3.2
LVOT min diameter, mm	22.5 ± 3.1
LVOT max diameter, mm	28.8 ± 3.5
LVOT area, mm^2^	511 ± 130
LVOT eccentricity index	0.78 ± 0.06
AV calcium score, AU	3364.6 ± 2236.0
**Comparison between CT and Echo**	
GOA_CT_-EOA_Echo_, cm^2^	0.07 ± 0.19
EOA_Echo_/GOA_CT_ ratio	0.94 ± 0.25
EOA derived severe AS, *n* (%)	147 (90.2)
GOA derived severe AS, *n* (%)	136 (83.4)
Concordant AS severity, *n* (%)	144 (88.3)
Discordant AS severity, *n* (%)	19 (11.7)

AS, aortic stenosis; AU, arbitrary unit; AV, aortic valve; E/e', the ratio of early diastolic mitral inflow velocity to the early diastolic mitral annular velocity; EOA, effective orifice area; GOA, geometric orifice area; LVEDD, left ventricular end-diastolic dimension; LVESD, left ventricular end-systolic dimension; LVEF, left ventricular ejection fraction; LV GLS, left ventricular global longitudinal strain; LA, left atrium; LV, left ventricle; LVOT, left ventricular outflow tract.

### EOA vs. GOA: Correlations with transaortic velocity and MPG

**Figure 3:**
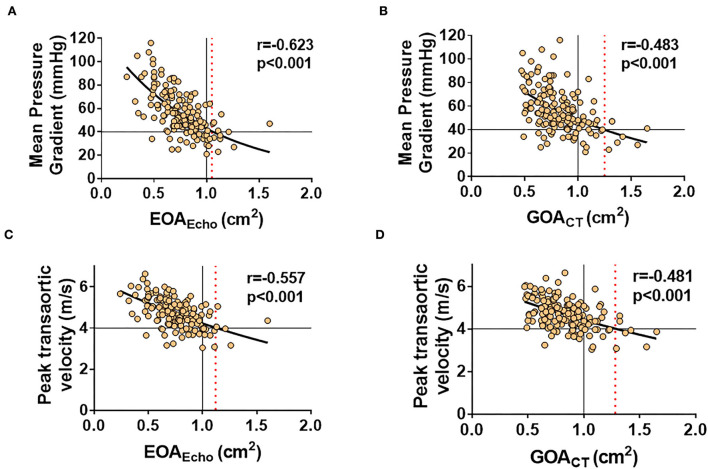
Correlations of EOA_Echo_ and GOA_CT_ to hemodynamic variables across the stenotic valve. MPG 40 mmHg corresponded to EOA_Echo_ of 1.05 cm^2^ **(A)** and GOA_CT_ of 1.25 cm^2^ **(B)**. The transaortic peak flow velocity of 4 m/s corresponded to EOA_Echo_ of 1.12 cm^2^ **(C)** and GOA_CT_ of 1.28 cm^2^ **(D)**, respectively.

Both EOA_Echo_ and GOA_CT_ showed modest correlations with transaortic MPG and peak velocity (all *p* < 0.001; Figure 3). The correlation coefficients between the two methods were not significantly different. The correlations were consistent according to combined AR or EF (Supplementary Figure 2). MPG 40 mmHg corresponded to EOA_Echo_ of 1.05 cm^2^ and GOA_CT_ of 1.25 cm^2^. The transaortic peak flow velocity of 4 m/s corresponded to EOA_Echo_ of 1.12 cm^2^ and GOA_CT_ of 1.28 cm^2^, respectively (Figure 3).

### EOA vs. GOA: Correlations with patient symptoms, biomarkers, and LV function

**Figure 4:**
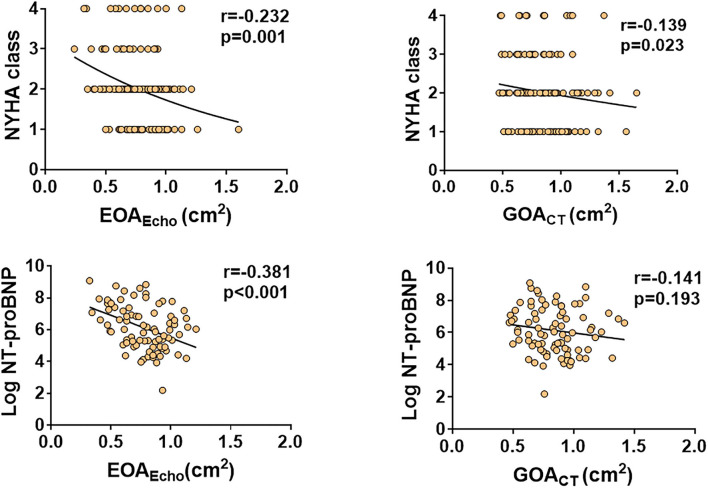
Correlations of EOA_Echo_ and GOA_CT_ to patient symptom status and biomarkers.

EOA_Echo_ showed weak correlations with NYHA class (*r* = −0.287, *p* < 0.001) and modest correlations with log-transformed NT-proBNP (*r* = −0.381, *p* = 0.001). GOA_CT_ showed weak correlations with NYHA class (*r* = −0.139, *p* = 0.023), but not with log-transformed NT-proBNP (*r* = −0.141, *p* = 0.193) (Figure 4). Table 3 shows correlations of AV area to functional echocardiographic parameters and biomarkers. EOA_Echo_ had better correlations with LA volume index, *E/e'*, LVEF, and LV GLS than GOA_CT_. These correlations were similar according to the LVEF subgroup (Supplementary Figure 3).

**Table 3:** Correlations of aortic valve area with cardiac function and biomarkers.

	**EOA**_**Echo**_ **, cm** ^**2**^		**GOA**_**CT**_ **, cm** ^**2**^		***p*** -value^*^
	***r***	***p*** **-value**	***r***	***p*** **-value**	
LV mass index, g/m^2^	−0.035	0.660	0.135	0.086	0.127
Relative wall thickness	−0.242	0.002	−0.382	< 0.001	0.166
LA volume index, ml/m^2^	−0.231	0.003	−0.006	0.935	0.040
E velocity, cm/s	−0.174	0.027	0.083	0.292	0.021
e' velocity, cm/s	0.131	0.096	0.188	0.017	0.602
E/e'	−0.236	0.003	−0.059	0.462	0.108
PASP, mmHg	−0.218	0.007	−0.020	0.804	0.081
LVEF, %	0.198	0.011	−0.052	0.511	0.024
|LV GLS|, %	0.205	0.009	0.002	0.984	0.066
Log NT-pro BNP, pg/ml	−0.381	< 0.001	−0.141	0.193	0.093
AV calcium score, AU	−0.027	0.746	0.051	0.533	0.502
	**Indexed EOA**_Echo_ **, cm** ^2^ **/m** ^2^		**Indexed GOA**_CT_ **, cm** ^2^ **/m** ^2^		***p*** **-value** ^*^
	***r***	***p*** **-value**	***r***	***p*** **-value**	
LV mass index, g/m^2^	−0.113	0.152	0.064	0.422	0.113
Relative wall thickness	−0.252	0.002	−0.379	< 0.001	0.208
LA volume index, ml/m^2^	−0.247	0.001	−0.006	0.936	0.028
E velocity, cm/s	−0.168	0.032	0.105	0.183	0.014
*e'* velocity, cm/s	0.158	0.045	0.211	0.007	0.624
*E/e'*	−0.236	0.003	−0.043	0.591	0.080
PASP, mmHg	−0.167	0.039	0.038	0.638	0.074
LVEF, %	0.289	< 0.001	0.039	0.618	0.021
|LV GLS|, %	0.337	< 0.001	0.141	0.072	0.062
Log NT–pro BNP, pg/ml	−0.394	< 0.001	−0.147	0.174	0.082
AV calcium score, AU	−0.206	0.011	−0.136	0.097	0.535

AU, arbitrary unit; AV, aortic valve; E, early diastolic mitral inflow; e', early diastolic mitral annular, E/e', the ratio of early diastolic mitral inflow velocity to early diastolic mitral annular velocity; GLS, global longitudinal strain; LA, left atrium; LV, left ventricle; LVEF, left ventricular ejection fraction; NT-proBNP, N-terminal pro brain natriuretic peptide; PASP, pulmonary artery systolic pressure.

* Comparison between EOA_Echo_ and GOA_CT_.

## Discussion

The main findings of the present study are as follows: (1) In bicuspid AS, there was a significant correlation between EOA_Echo_ and GOA_CT_, but the degree of correlation was modest. (2) Both EOA_Echo_ and GOA_CT_ had modest correlations with conventional hemodynamic parameters determining AS severity. (3) Transaortic MPG of 40 mmHg corresponds to EOA_Echo_ of 1.05 cm^2^ and GOA_CT_ of 1.25 cm^2^. (4) EOA_Echo_ had better correlations with echocardiographic parameters indicating LV systolic and diastolic function, regardless of EF status, than GOA_CT_. These findings suggest that GOA_CT_ is an attractive alternative to EOA_Echo_ due to the direct measurement of stenotic valve area, but diagnostic criteria for severe AS should be applied as a higher cutoff compared to EOA_Echo_, and there are limitations in that the correlation with LV functional alteration is less than that of EOA_Echo_. To the best of our knowledge, this is the first study to investigate the utility of GOA_CT_ on bicuspid AS assessment and comprehensive comparison between EOA_Echo_ and GOA_CT_ in bicuspid AS.

### Assessment of AS severity

According to current guidelines, severe AS is defined as EOA_Echo_ of <1.0 cm^2^, transaortic peak velocity of > 4.0 m/s, or MPG of > 40 mmHg (11, 23). Doppler echocardiography remains the standard method for assessing AS severity. Cardiac CT is particularly useful in patients with poor acoustic windows and provides additional anatomical information about both adjacent structures and the aortic valve itself for planning surgical or transcatheter aortic valve replacement (7, 24, 25). In tricuspid AS, EOA_Echo_ showed a significant correlation with GOA_CT_ (4). However, there were some discrepancies in AS severity between echocardiographic parameters and GOA measurement by cardiac CT (5, 24).

In bicuspid AS, the functional severity measured by Doppler echocardiography tends to be more severe than that of tricuspid AS because of greater jet eccentricity and less pressure recovery, resulting in higher energy loss (3, 26). As a result, it has been reported that there was a greater discrepancy between EOA_Echo_ and GOA_CT_ in bicuspid AS compared with tricuspid AS (3). However, no study had confirmed the associations with hemodynamic variables of the AV and LV or evaluated clinical significance in patients with bicuspid AS. Therefore, we believe that the modest correlation observed between EOA_Echo_ and GOA_CT_, the fact that valve area measured by both methods has good correlations with AV hemodynamic variables, and the stronger association of EOA_Echo_ with LV functional variables observed will help clinicians better understand the AV area evaluated by multimodal imaging.

A previous study that reported inconsistencies of echocardiographic criteria for the grading of AS showed that MPG 40 mmHg corresponds to EOA_Echo_ of 0.75 cm^2^ and peak velocity of 4.0 m/s to EOA_Echo_ of 0.82 cm^2^ (5). In the present study, we observed higher corresponding EOA_Echo_ and GOA_CT_ values with conventional echocardiographic parameter thresholds than previously reported values. Since the previous study was not limited to bicuspid AS, the majority of patients must have had tricuspid AS (5). Therefore, the larger EOA_Echo_ corresponding to MPG 40 mmHg or peak velocity of 4.0 m/s is consistent with the results of previous studies showing higher gradients in bicuspid AS than in tricuspid AS (8, 27, 28). To the best of our knowledge, the present study is the first to provide a threshold for severe bicuspid AS assessed by cardiac CT. Further studies are needed to elucidate the prognosis of bicuspid severe AS determined by cardiac CT.

### Differences in valve area in multimodal imaging

A previous study conducted head-to-head comparisons of valve area calculation by Doppler echocardiography and CT in 269 patients with calcified AS (4). They found that AV area measured by CT resulted in higher values than EOA_Echo_ and had higher cutoff values (1.2 cm^2^) to predict mortality under medical treatment. However, AVA_CT_ did not improve concordance for AS grading or outcome prediction. Although in the present study, we did not examine clinical outcomes according to AV area, we evaluated clinical significance on LV alteration as we observed better correlations of EOA_Echo_ with estimated LV filling pressure, LA volume index, and LV global longitudinal strain than GOA_CT_. As LV GLS and the extent of cardiac damage associated with AS have important prognostic implications, our results indicate that EOA_Echo_ is meaningful as a gold standard for AV area calculation, similar to the results of a previous study of tricuspid AS (4, 29, 30). In addition, we observed higher corresponding values of GOA_CT_ to MSPG > 40 mmHg, which indicates severe AS, than EOA_Echo_. This suggests that it is desirable to use a larger cutoff value for GOA_CT_ when using GOA_CT_ in cases where EOA_Echo_ assessment is unreliable or impossible.

### Study limitations

First, this study was a retrospective cross-sectional study from a single tertiary center and thus has intrinsic limitations. Clinical events and follow-up data of patients were not analyzed in this study. However, we provide clinically important information regarding AV area calculation from two different imaging modalities in patients with symptomatic bicuspid AS. Second, data to determine invasive hemodynamic data were not available in the present study. However, invasive hemodynamic data are rarely used to assess AS in clinical practice and current guidelines recommend the use of cardiac catheterization in poor echocardiographic windows and low-flow status. Our study subjects had a mean stroke volume index of 48.4 ± 10.2 ml/m^2^, which means most of our study population had normal-flow AS. Third, because this study was conducted among patients with severe bicuspid AS who were referred to surgery, the results of this study cannot be equally generalized to a wider patient range, that is, mild-to-moderate patients with AS or asymptomatic patients. Fourth, the present study was cross-sectional in design, and extensive cardiac damage might have been present before AS. However, bicuspid AS occurs in relatively younger and healthier patients than tricuspid AS (31). We believe that most of our study subjects had AS-associated structural abnormalities. Fifth, the study population underwent Doppler echocardiography and CT within 3 months, not simultaneously. The time difference between echocardiography and CT might result in discordance between hemodynamic parameters and GOA_CT_. In the future, well-designed comparisons are warranted to clarify our findings and determine the clinical impact of GOA_CT_ in bicuspid AS. Sixth, not all patients underwent NT-proBNP tests, resulting in potential selection bias. Further prospective studies of the prognostic impact of EOA_Echo_ and GOA_CT_ are needed.

## Conclusion

GOA_CT_ can be used to evaluate the severity of bicuspid AS. The threshold for GOA_CT_ for diagnosing severe AS should be higher than that for EOA_Echo_. However, EOA_Echo_ is still the method of choice because EOA_Echo_ showed better correlations with clinical and functional variables than GOA_CT_. These findings suggest that EOA_Echo_ should be the method of choice to assess bicuspid AS severity, but GOA_CT_ is an effective alternative in patients with poor echocardiographic image quality.

## Data availability statement

The raw data supporting the conclusions of this article will be made available by the authors, without undue reservation.

## Ethics statement

The studies involving human participants were reviewed and approved by the Institutional Review Board of Severance Hospital. Written informed consent for participation was not required for this study in accordance with the national legislation and the institutional requirements.

## Author contributions

KK, YK, and CS contributed to conception and design of the study. KK, SL, JS, IC, G-RH, and J-WH organized the database. KK performed the statistical analysis and wrote the first draft of the manuscript. KK, YS, YK, and CS wrote sections of the manuscript. All authors contributed to manuscript revision, read, and approved the submitted version.
