# Supplementary material for: Assessment of aortic valve area on cardiac computed tomography in symptomatic bicuspid aortic stenosis: Utility and differences from Doppler echocardiography
Source: Front Cardiovasc Med. 2022 Dec 19;9:1035244. doi: 10.3389/fcvm.2022.1035244 (PMC9807240; doi:10.3389/fcvm.2022.1035244)
Supplement: Supplementary file 1 [file Data_Sheet_1.docx]

**Supplementary Figure 1.** Geometric orifice area measurement from cardiac computed tomography in patient with type 0 (A), and other types of bicuspid aortic valve (B).

**
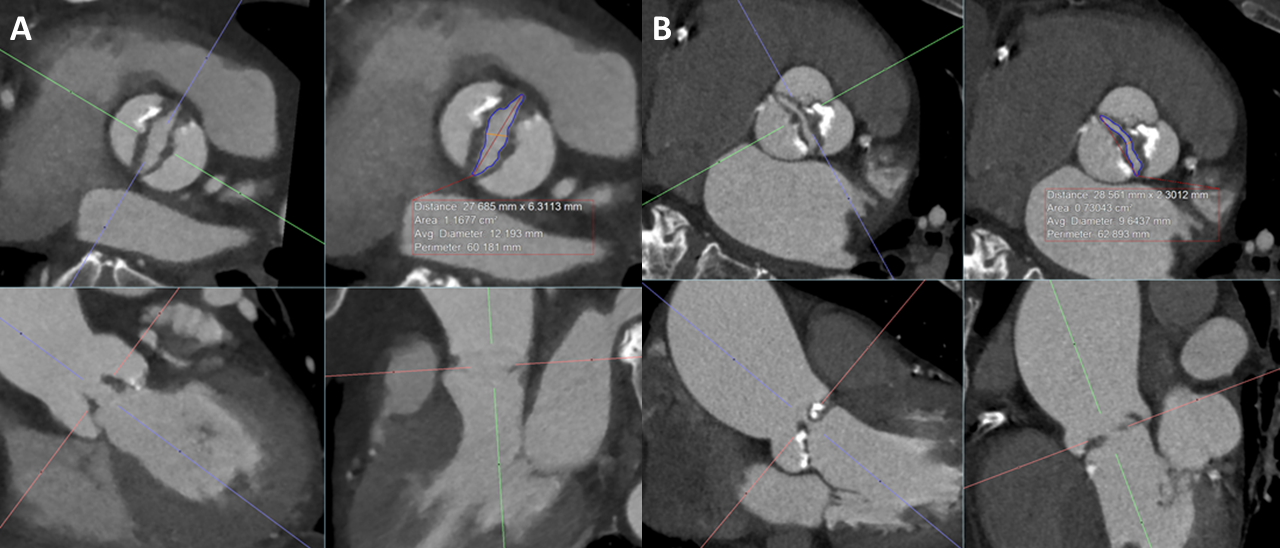
**

**Supplementary Figure 2.** Correlations of EOA_Echo_ and GOA_CT_ to mean transaortic pressure gradient, according to combined significant aortic regurgitation and left ventricular ejection fraction.


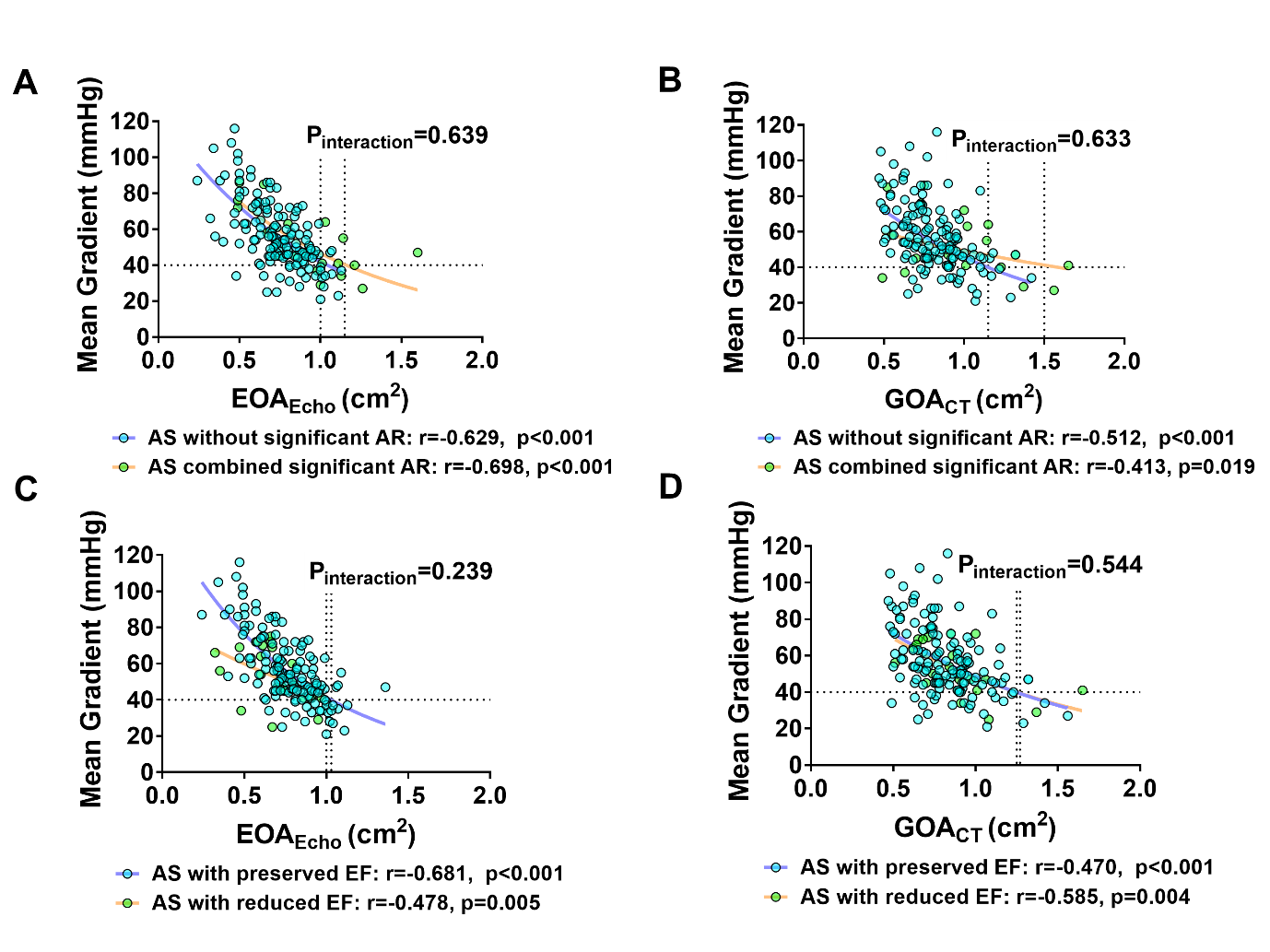


**Supplementary Figure 3.** Correlations of EOA_Echo_ to E/e’ and |LV GLS|, according to left ventricular ejection fraction.

_
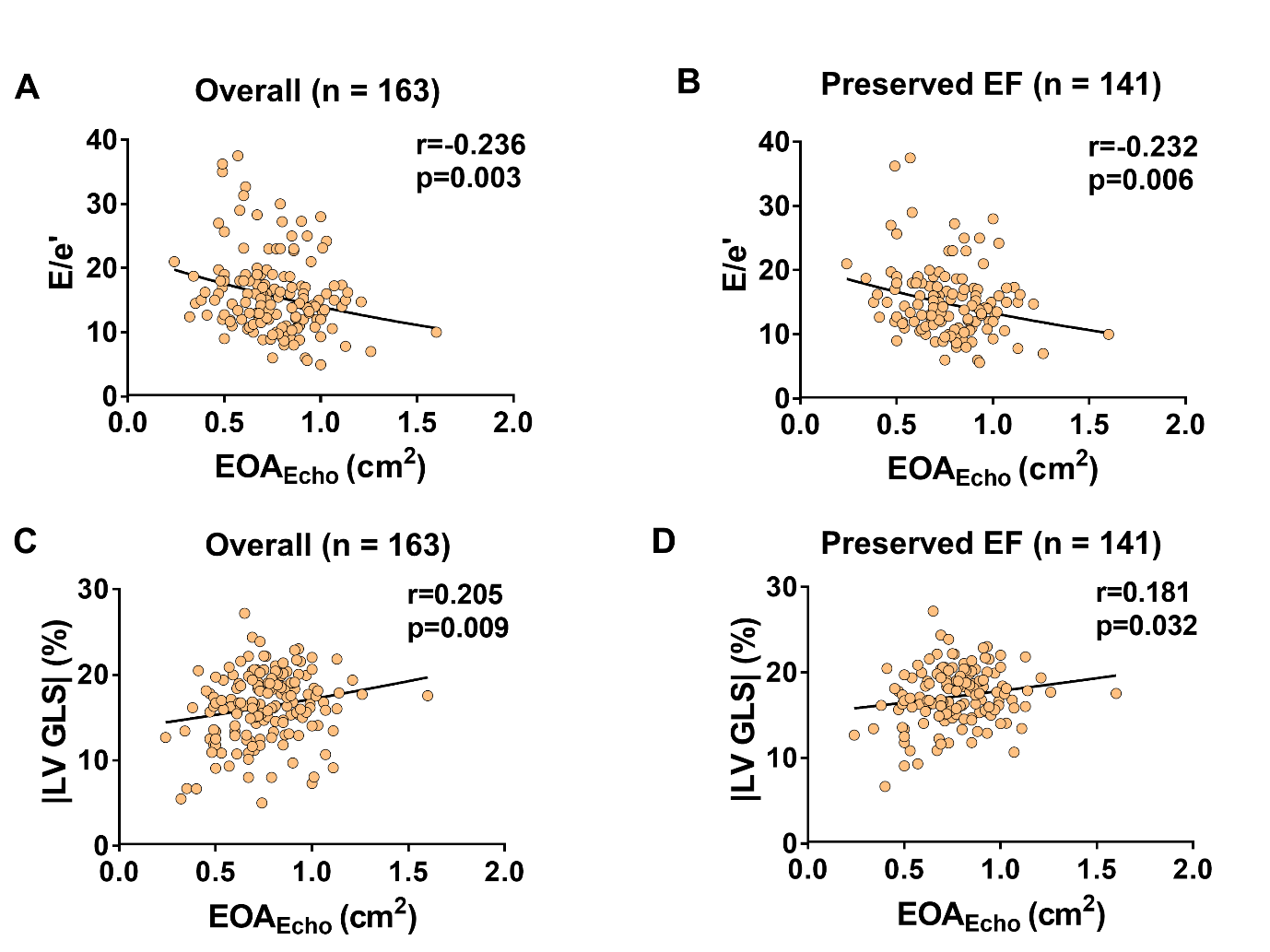
_

**Supplementary Table 1. Baseline characteristics of study population according to concordance aortic stenosis degree.**

|  | **Concordant N=144** | **Discordant N=19** | | **P value** | |  |
| --- | --- | --- | --- | --- | --- | --- |
| Age, years | 64.30 ± 9.48 | 64.11 ± 11.01 | | 0.935 | |  |
| Male sex, n (%) | 79 (54.9) | 13 (68.4) | | 0.382 | |  |
| Body mass index, g/m^2^ | 24.53 ± 5.22 | 25.36 ± 3.22 | | 0.336 | |  |
| Hypertension, n (%) | 76 (52.8) | 12 (63.2) | | 0.543 | |  |
| Diabetes mellitus, n (%) | 30 (20.8) | 6 (31.6) | | 0.443 | |  |
| Coronary artery disease, n (%) | 17 (11.8) | 2 (10.5) | | >0.999 | |  |
| Dyslipidemia, n (%) | 45 (31.3) | 6 (31.6) | | >0.999 | |  |
| Chronic kidney disease, n (%) | 3 (2.1) | 1 (5.3) | | >0.958 | |  |
| Atrial fibrillation, n (%) | 19 (13.2) | 2 (10.5) | | >0.999 | |  |
| Prior cerebrovascular accident, n (%) | 1 (0.7) | 0 (0) | | >0.999 | |  |
| Systolic blood pressure, mmHg | 121.4 ± 13.0 | 128.0 ± 13.6 | | 0.043 | |  |
| Diastolic blood pressure, mmHg | 76.3 ± 10.4 | 75.2 ± 12.2 | | 0.656 | |  |
| Pulse pressure, mmHg | 45.1 ± 9.9 | 52.8 ± 16.1 | | 0.057 | |  |
| Bicuspid AV morphology | | |  | |  | |
| Type 1 (RCC+LCC), n (%) |  |  | |  | |  |
| Type 2 (RCC+NCC), n (%) |  |  | |  | |  |
| Type 3 (LCC+NCC), n (%) |  |  | |  | |  |
| Type 0 (No raphe), n (%) |  |  | |  | |  |
| Log NT-proBNP, pg/ml | 6.14 ± 1.50 | 6.13 ± 1.62 | | 0.972 | |  |
| NYHA class, n (%) |  |  | | 0.002 | |  |
| I | 35 (24.3) | 8 (42.1) | |  | |  |
| II | 80 (55.6) | 5 (26.2) | |  | |  |
| III | 21 (14.6) | 1 (5.3) | |  | |  |
| IV | 8 (5.6) | 5 (26.3) | |  | |  |
| Type of surgery performed |  |  | |  | |  |
| Aortic valve replacement or aorta replacement | 132 (91.7) | 17 (89.5) | | >0.999 | |  |
| Aortic valve replacement | 128 (89.0) | 17 (89.5) | | >0.999 | |  |
| Aorta replacement | 38 (27.1) | 4 (23.5) | | 0.978 | |  |

RCC, right coronary cusp; LCC, left coronary cusp; NCC, non-coronary cusp; NT-proBNP, N-terminal pro brain natriuretic peptide; NYHA, New York Heart Association
